# Inhibition of NLRP1 inflammasome improves autophagy dysfunction and Aβ disposition in APP/PS1 mice

**DOI:** 10.1186/s12993-023-00209-8

**Published:** 2023-04-13

**Authors:** Xuewang Li, Han Zhang, Liu Yang, Xianan Dong, Yuli Han, Yong Su, Weiping Li, Weizu Li

**Affiliations:** 1 Department of Pharmacology, Basic Medicine College, Key Laboratory of Anti-Inflammatory and Immunopharmacology, Ministry of Education, Anhui Medical University, Hefei, 230032 China; 2 Department of Pharmacy, The First Affiliated Hospital of Anhui Medical University, Hefei, 230032 Anhui China; 3 Anqing Medical and Pharmaceutical College, Anqing, 246052 Anhui China

**Keywords:** Alzheimer's disease, NLRP1 inflammasome, AMPK/mTOR, Autophagy, APP/PS1 mice

## Abstract

Increasing evidence has shown that the NOD-like receptor protein 1 (NLRP1) inflammasome is associated with Aβ generation and deposition, which contributes to neuronal damage and neuronal-inflammation in Alzheimer's disease (AD). However, the specific mechanism of NLRP1 inflammasome in the pathogenesis of AD is still unclear. It has been reported that autophagy dysfunction can aggravate the pathological symptoms of AD and plays an important role in regulating Aβ generation and clearance. We hypothesized that NLRP1 inflammasome activation may induce autophagy dysfunction contributing to the progression of AD. In the present study, we observed the relationship between Aβ generation and NLRP1 inflammasome activation, as well as AMPK/mTOR mediated-autophagy dysfunction in WT 9-month-old (M) mice, APP/PS1 6 M and APP/PS1 9 M mice. Additionally, we further studied the effect of NLRP1 knockdown on cognitive function, Aβ generation, neuroinflammation and AMPK/mTOR mediated autophagy in APP/PS1 9 M mice. Our results indicated that NLRP1 inflammasome activation and AMPK/mTOR mediated-autophagy dysfunction are closely implicated in Aβ generation and deposition in APP/PS1 9 M mice, but not in APP/PS1 6 M mice. Meanwhile, we found that knockdown of NLRP1 significantly improved learning and memory impairments, decreased the expressions of NLRP1, ASC, caspase-1, p-NF-κB, IL-1β, APP, CTF-β, BACE1 and Aβ_1-42_, and decreased the level of p-AMPK, Beclin 1 and LC3 II, and increased the level of p-mTOR and P62 in APP/PS1 9 M mice. Our study suggested that inhibition of NLRP1 inflammasome activation improves AMPK/mTOR mediated-autophagy dysfunction, resulting in the decrease of Aβ generation, and NLRP1 and autophagy might be important targets to delay the progression of AD.

**Supplementary Information:**

The online version contains supplementary material available at 10.1186/s12993-023-00209-8.

## Background

Alzheimer's disease (AD), the most typical neurodegenerative disease [1, 2], is characterized clinically by a progressive and irreversible loss of cognitive functions, pathological synaptic dysfunction and neuronal damage, as well as the production of extracellular deposits of β-amyloid (Aβ) peptides [3]. Especially, Aβ deposition-formed plaque is one of critical neuropathological hallmarks of AD, which is closely correlated to synaptic dysfunction and neuronal damage in AD [4]. It is well known that Aβ is generated from the amyloid precursor protein (APP) cleavaged sequentially by β-secretase and γ-secretase [5]. However, the mechanisms which regulate Aβ generation and deposition are still incompletely understood. It has been reported that the levels of cytokines, such as tumor necrosis factor alpha (TNF-α) and interleukin-6 (IL-6) in serum and cerebrospinal fluid of patients with AD and mild cognitive impairment (MCI), increase steadily during the time of MCI to AD conversion, suggesting that neuroinflammation may closely correlate with progression of AD [6]. Furthermore, increasing studies have suggested that interaction of Aβ production and neuroinflammation can converge to promote the pathological injury associated with AD, and inhibiting neuroinflammation looks like a promising treatment for restraining further development of AD [7, 8]. However, the precise mechanism of how neuroinflammation regulates Aβ deposition remains unclear.

Neuroinflammation is generally considered to be activated by microglia, which participates in the clearance of Aβ and the secretion of neurotoxic molecules to induce neurodegeneration [9]. However, anti-inflammation treatment through microglia did not get the expected effects for AD patients, suggesting that other inflammation pathways might involve in progression of AD [10]. It is well known that degenerated neurons, inflammation and synapse loss are not only the common responses initiated by neurons but also the best markers of AD [11]. The NOD-like receptor protein 1 (NLRP1) is a critical component of the inflammasome, which is a multiprotein oligomer that contributes to inflammation and appears to be expressed ubiquitously in many tissues, and high level of NLRP1 is also expressed in the brain, particularly in neurons [12, 13]. Growing evidence has reported that the NLRP1 inflammasome closely correlates with AD. For example, the NLRP1 immunopositive neurons in AD brains were increased by 25–30 folds compared with non-AD brains [13]. Similarly, inhibiting the expression of NLRP1 could reduce the Aβ deposition in the brain of AD model mice [14]. Additionally, our previous study found that inhibiting the NLRP1 inflammasome could prevent hippocampal neuron damage induced by glucocorticoid exposure [15]. However, the exact mechanism of NLPR1 inflammasome in regulating Aβ production remains unclear.

Autophagy is a highly conserved homeostasis process in which cytoplasmic macromolecules and damaged organelles are transported to the lysosome for degradation [16]. Generally, the autophagy is largely activated in cellular starving condition. Today it has been reported that autophagy can be induced by diverse stimulations, such as reactive oxygen species (ROS), hypoxia impairments, subcellular organelle damage, and protein aggregation [17]. Increasing studies have shown that autophagy dysfunction can aggravate the pathological symptoms of AD [18, 19]. Autophagy plays an important role in regulating Aβ generation and clearance [20]. Although Aβ is considered to be generated in endoplasmic reticulum (ER), lysosome and Golgi apparatus, it has been reported that Aβ is produced through cleavage of APP in the autophagosomes after autophagy activation [21, 22]. Moreover, recent studies have suggested that the autophagy-lysosome pathway also plays an important role in Aβ peptides clearance [23]. Additionally, a recent study reported that the APP/PS1 mice exhibited excessive autophagy and lysosomal dysfunction, and that was accompanied by cognitive impairment and neuronal injury in hippocampus CA1 area [24]. These studies suggested that autophagy might be closely correlated with progression of AD, but it is still unknown whether NLRP1 inflammasome regulates autophagy activation in AD.

In this study, we hypothesized that excessive APP and Aβ generation might activate NLRP1 inflammasome and induce autophagy dysfunction, which engulfs and processes APP to increase Aβ generation in APP/PS1 mice, and knockdown of NLRP1 might improve autophagy dysfunction and decrease Aβ deposition to delay progression of AD. In the present study, we observed the changes of autophagy and NLRP1 inflammasome in 6-month-old (M) and 9 M APP/PS1 mice. Then, we further studied the effect of NLRP1 knockdown on AMPK/mTOR pathway-mediated autophagy response, cognitive dysfunction and Aβ production in APP/PS1 mice.

## Materials and methods

### Animals and treatment

The APP/PS1 double transgenic mice were purchased from the Model Animal Research Center of Nanjing University and were bred in the animal center of Anhui Medical University. Mice were housed in standard laboratory conditions with free access to standard food and water (12-h light/dark cycle). Male 6 months (M) and 9 M APP/PS1 mice were used in the experiment, and male 9 M WT littermates were used as controls. The experimental protocols were approved by the animal ethics committee of Anhui Medical University (LLSC20160183).

To study the effect of NLRP1-siRNA on APP/PS1 mice, six months old male APP/PS1 mice were divided into 3 groups (n = 8): normal saline (NS) control, lentivirus (LV)-scramble negative control, LV-NLRP1-siRNA-treated group. The negative lentivirus vector sequence is 5'-TTC TCC GAA CGT GTC ACG T-3', NLRP1-siRNA is 5'-GCT CTT CTT CTT CTT CTA ACA-3' (1 × 10^7^ TU/ml, Shanghai GenePharma Co., China), which we have reported to significantly decrease NLRP1 expression in our previous study [15]. NS, control lentivirus or NLRP1-siRNA lentivirus (2 μl each) were stereotactically injected into the left cerebral lateral ventricles as described in the previous study (coordinates: 0.6 mm posterior to bregma; lateral: −1.5 mm; dorsoventral: 1.7 mm) [25]. The mice were maintained for 12 weeks after the injection of the lentivirus. After the behavior test, the mice were killed by cervical dislocation and the brains were quickly removed for other experiments. The experimental flow chart is shown in Additional file 1: Figure S1.

### Open field test (OFT)

The OFT system (Shanghai Biotechnology Co., Ltd.) includes a video-tracked cage (60 × 60 × 50 cm) and ANY-maze behavior tracking software (Stoelting Co., Wood Dale, IL, USA). The cage was divided into nine squares by two horizontal lines and two vertical lines as described in a previous study [26]. For OFT, each mouse was placed in the cage for 2 min to adapt to the environment. Then the ANY-maze behavioral tracking software was used to record the motor activity for 3 min. The moving distance (MD, m), the mean movement speed (MMS, m/s), the number of lines crossed (NLC), and the number of standing up (NSU) were examined to indicate the motor activity behavior. The OFT was performed every 4 weeks to evaluate the changes of motor activity.

### Morris water maze (MWM) test

MWM is an important method to detect learning and memory impairments [27]. The test pool (120 cm in diameter and 60 cm high) filled with water was divided into four quadrants. There was a hidden escape platform (9 cm in diameter) that was submerged 1 cm below the surface of the water in the third quadrant. The MWM test includes four consecutive daily training trials and the fifth day of exploration trials as described in our previous study [28]. The mice were individually placed into the tank and were allowed to circumnavigate the pool to search the escape platform for four trials (60 s per trial) per day. The mean escape latency (MEL, s) of the four trials each day was recorded to indicate the learning performance. On the fifth day, the platform was removed from the pool, and each mouse performed a 60 s swimming probe trial. The latency of first entry to the platform (LFP, s), the swimming time in the quadrant of the platform (STP, s), and the number of crossing the platform (NCP) were recorded to indicate the memory results.

### Histological examination

After the MWM test, the mice (n = 4) were sacrificed by cervical dislocation and the brains were removed and immersed in 4% paraformaldehyde to fix the brains. The brain tissues were embedded in paraffin and sliced into 5 μm sections using a microtome (Leica, Nussloch, Germany). The sections were stained with hematoxylin and eosin (H&E) to examine the pathological changes in the cortex by intelligent tissue slice imaging analysis system platform (Pannoremic MIDI, 3DHISTECH, Hungary). Three random fields (400×) in the same cortex region from 4 mice in each group were used to evaluate the pathological damage of neurons by a pathologist double-blindly. Besides, Nissl staining is often used to identify neuronal damage [29]. We further performed the Nissl staining to study neuronal pathological alterations. The sections (n = 4) were treated with Nissl staining solution (Beyotime Biotechnology, China) for 10 min and were differentiated with 95% alcohol. Then the sections were observed and photographed by intelligent tissue slice imaging analysis system platform (Pannoremic MIDI, 3DHISTECH, Hungary). The density of the Nissl bodies was analyzed double-blindly from three random fields (400×) in the same cortex and hippocampal CA1 regions from 4 mice in each group by using the Image-Pro Plus 6.0 automatic analysis system to assess the change of Nissl bodies.

### Immunofluorescence

The paraffin sections (n = 4) were used to perform immunofluorescence. Briefly, the sections were deparaffinized and hydrated. Then the sections were permeabilized with Triton X-100 (0.25%) for 30 min, followed by blocking with BSA (1%) for 60 min. Then the sections were incubated with primary antibody of Aβ_1-42_ (1:150, Bioss Technology, bs-0076R) overnight at 4 °C. The next day, the sections were incubated with secondary antibody which was conjugated to Rhodamine (1:200, ZSGB-BIO). Then the sections were mounted by using an anti-fade medium and examined by intelligent tissue slice imaging analysis system platform (Pannoremic MIDI, 3DHISTECH, Hungary). The fluorescence density was analyzed double-blindly from three random fields (400×) of the same cortex region from 4 mice in each group by using Image-Pro Plus 6.0 automatic analysis system to indicate the changes of Aβ_1-42_ expression.

## Thioflavin-S staining

Paraffin sections (n = 4) were deparaffinized with xylene and were hydrated by graded alcohol. Then the sections were immersed in 1% Thioflavin-S (HY-D0972, MedChemExpress USA) for 5 min, hydrated in 70% ethanol and washed twice with PBS (5 min each time). After staining with Hochest 33258 for 5 min, the slides were sealed with an anti-fade medium. The intelligent tissue slice imaging and analysis system platform (Pannoremic MIDI, 3DHISTECH, Hungary) was used to scan the images. The fluorescence density was analyzed double-blindly from three random fields (400 ×) of the same cortex region from 4 mice in each group by using Image-Pro Plus 6.0 automatic analysis system to evaluate the changes of Thioflavin-S positive areas, which indicate the amyloid deposits.

### Immunoblot analysis

The hippocampus and cortex tissues were homogenized in RIPA lysis (Beyotime Biotechnology, Shanghai, China) with protease and phosphatase inhibitors. Total protein was extracted by using a cryogenic tissue grinder (Shanghai Jingxin Industrial Development Co., Ltd) at 4 °C, 60 Hz for the 50 s. The BCA Protein Assay Kit (Beyotime Biotechnology, Shanghai, China) was used to determine protein concentration. The samples (n = 4) were separated by SDS-PAGE (12%) and transferred onto PVDF membranes (Millipore, Bedford, MA, USA). Afterward, membranes were blocked in 5% non-fat milk in Tween20 TBS (TBST) for 1 h at room temperature, followed with the primary antibodies (Additional file 5: Table S1**)** overnight at 4 °C. The next day, membranes were washed and incubated with a horseradish peroxidase-conjugated secondary antibody (ZSGB-BIO, ZF-2301, 1:10,000) for 1 h at room temperature. After washing three times with TBST, the proteins were visualized by an enhanced chemiluminescent reagent (Amersham Biosciences, UK). The bands were obtained by using a Chemi Q4800 mini imaging system (Shanghai Bioshine Technology, Shanghai, China). The density of the protein band was measured using Image J 6.0. The density of the target band was normalized to β-actin. The relative density of target protein over WT or NS control was used to indicate the changes of target proteins.

### Quantitative real-time PCR (q-PCR) analysis

For the q-PCR analysis, total RNA was extracted from the hippocampus and cortex tissues (n = 4) with TRIzol reagent (Invitrogen Co., USA) according to the instructions as described previously [30]. The first-strand cDNA was synthesized from total RNA with PrimeScript™ Reverse Transcriptase (Takara Bio, RR037A) according to the manufacturer’s protocol. Quantitative real-time PCR analyses for mRNAs of NLRP1, ASC, Caspase-1, IL-1β, APP, BACE1, NCSTN and β-actin were performed with SYBR^®^Premix Ex Taq™II RTPCR kits (Takara Bio, RR820A). The mRNA level of β-actin was used as an internal control. Primers are listed in Additional file 6: Table S2. PCR was performed at 95 °C for 30 s, followed by 40 cycles of amplification at 95 °C for 5 s, 60 °C for 30 s with Real-time PCR System (BIO-RAD, CFX96). The fluorescent signals were collected during the extension phase, CT values of the sample were calculated, and transcript levels were analyzed by 2^−ΔΔCT^ method. The q-PCR was repeated three times.

### Statistical analysis

All data are expressed as mean ± SD. Statistical analysis was performed using GraphPad Prism 8.0 software. The four-day training results of MWM and the data of OFT were analyzed by repeated-measures two-way analysis of variance (ANOVA) and other results were analyzed by one-way ANOVA. Tukey’s multiple-comparison test was used to compare the differences between groups. Statistical significance was defined as *P* < 0.05.

## Results

### Effects of aging on Aβ generation and neuronal damage in APP/PS1 mice

**Fig. 1:**
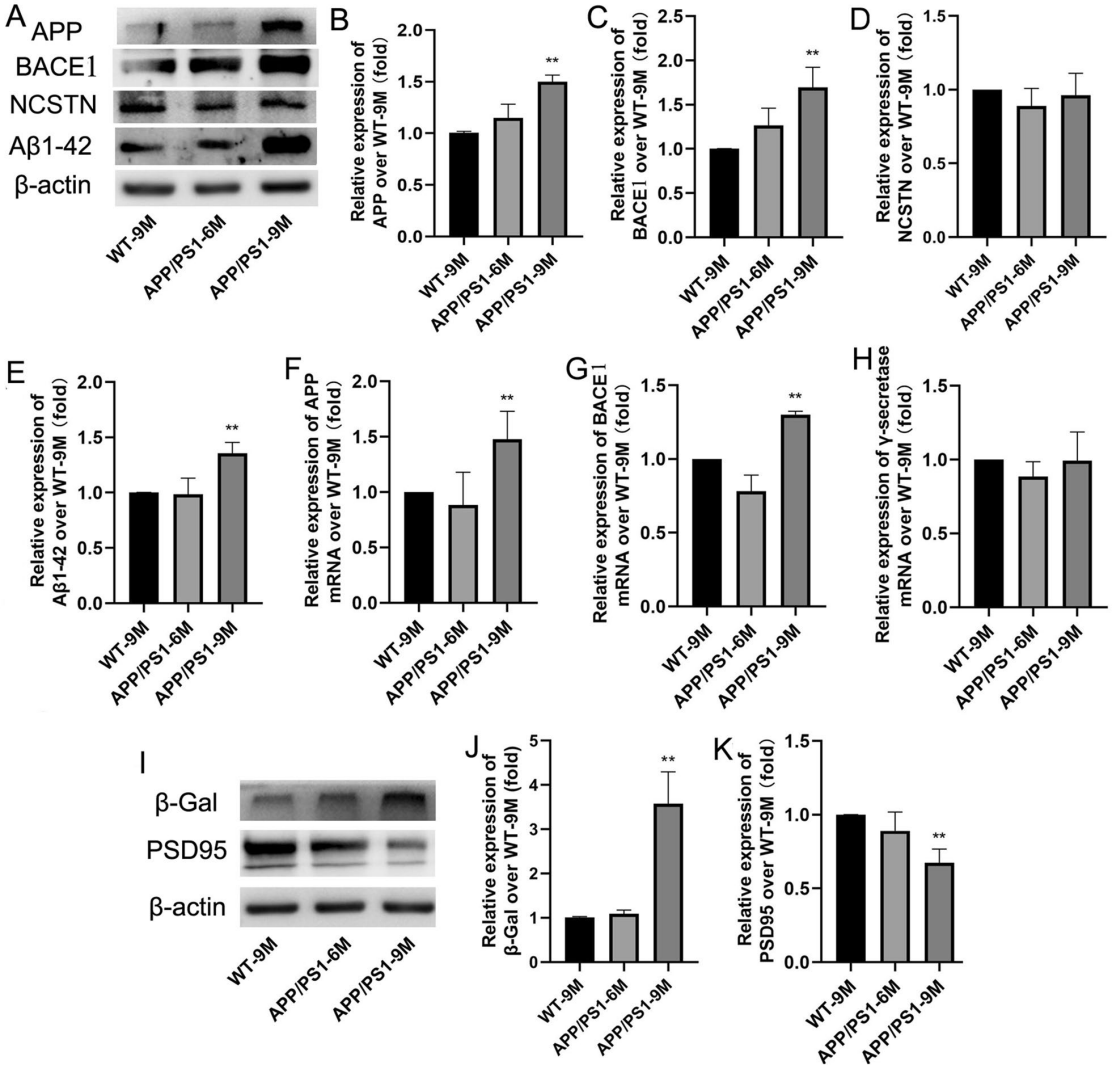
Effects of aging on Aβ generation and neuronal damage in APP/PS1 mice. **A** The bands of APP, BACE1, NCSTN, Aβ_1-42_ and β-actin (Western blot); **B** The relative expression of APP over WT-9 M; **C** The relative expression of BACE1 over WT-9 M; **D** The relative expression of NCSTN over WT-9 M; **E** The relative expression of Aβ_1-42_ over WT-9 M. **F** The relative mRNA expression of APP over WT-9 M (q-PCR); **G** The relative mRNA expression of BACE1 over WT-9 M (q-PCR); **H** The relative mRNA expression of NCSTN over WT-9 M (q-PCR). **I** The bands of β-gal, PSD95 and β-actin (Western blot); **J** The relative expression of β-gal over WT-9 M; **K** The relative expression of PSD95 over WT-9 M. Results are expressed as mean ± SD. n = 4. *P < 0.05, **P < 0.01 vs WT-9 M group

To observe the effects of aging on Aβ generation, we detected the expressions of APP, BACE1, NCSTN and Aβ_1-42_ in 6 M and 9 M APP/PS1 mice. The immunoblotting results showed that the expressions of APP, BACE1, NCSTN and Aβ_1-42_ had no significant increase in APP/PS1-6 M mice compared with the WT-9 M (Fig. 1A–E). While in APP/PS1-9 M mice, except for NCSTN, the expressions of APP, BACE1 and Aβ_1-42_ were significantly increased (Fig. 1B: F(2,9) = 27.58; Fig. 1C: F(2,9) = 39.52; Fig. 1E: F(2,9) = 29.33;* P* < 0.01). Meanwhile, we also detected the mRNA levels of APP, BACE1 and NCSTN by q-PCR. The results showed that, similar to the proteins, the mRNA levels of APP and BACE1 were also significantly increased in APP/PS1-9 M mice (Fig. 1F: F(2,6) = 5.899; Fig. 1H: F(2,6) = 48.84; *P* < 0.01).

The senescence-associated β-galactosidase (β-gal) is often measured to evaluate the aging-related neuronal damage [31] and postsynaptic density protein 95 (PSD95) is crucial for synaptic plasticity and is widely accepted as a regulatory protein that can regulate synapse function [32]. To evaluate the effect of aging on neuronal damage in APP/PS1 mice, we further detected the changes of β-gal and PSD95 in 6 M and 9 M APP/PS1 mice. The results showed that compared with WT-9 M mice, APP/PS1-6 M mice had no significant changes in β-gal and PSD95 (Fig. 1I–K). While in APP/PS1-9 M mice, the β-gal expression was significantly increased (Fig. 1I and J; F(2,9) = 36.83;* P* < 0.01) and the PSD95 expression was significantly decreased (Fig. 1I and K; F(2,9) = 13.36;* P* < 0.01). These data suggested that the Aβ generation is significantly increased and the aging-related neuronal damage is significantly accelerated in APP/PS1-9 M mice.

Meanwhile, we also confirmed the pathological changes and Aβ deposition in cortex by H&E staining and Thioflavin-S staining. The results indicated that there were no significant pathological changes and there was only fewer Aβ deposition in the cortex of APP/PS-6 M mice compared with WT-9 M mice (Additional file 2: Fig. S2 A and B). While in APP/PS1-9 M mice, the pyknotic cells around Aβ plaques (black arrows) and Aβ deposition (red arrows) were significantly increased in cortex of brain (Additional file 2: Fig. S2A and B).

### Effects of aging on NLRP1 inflammasome expression in the APP/PS1 mice

**Fig. 2:**
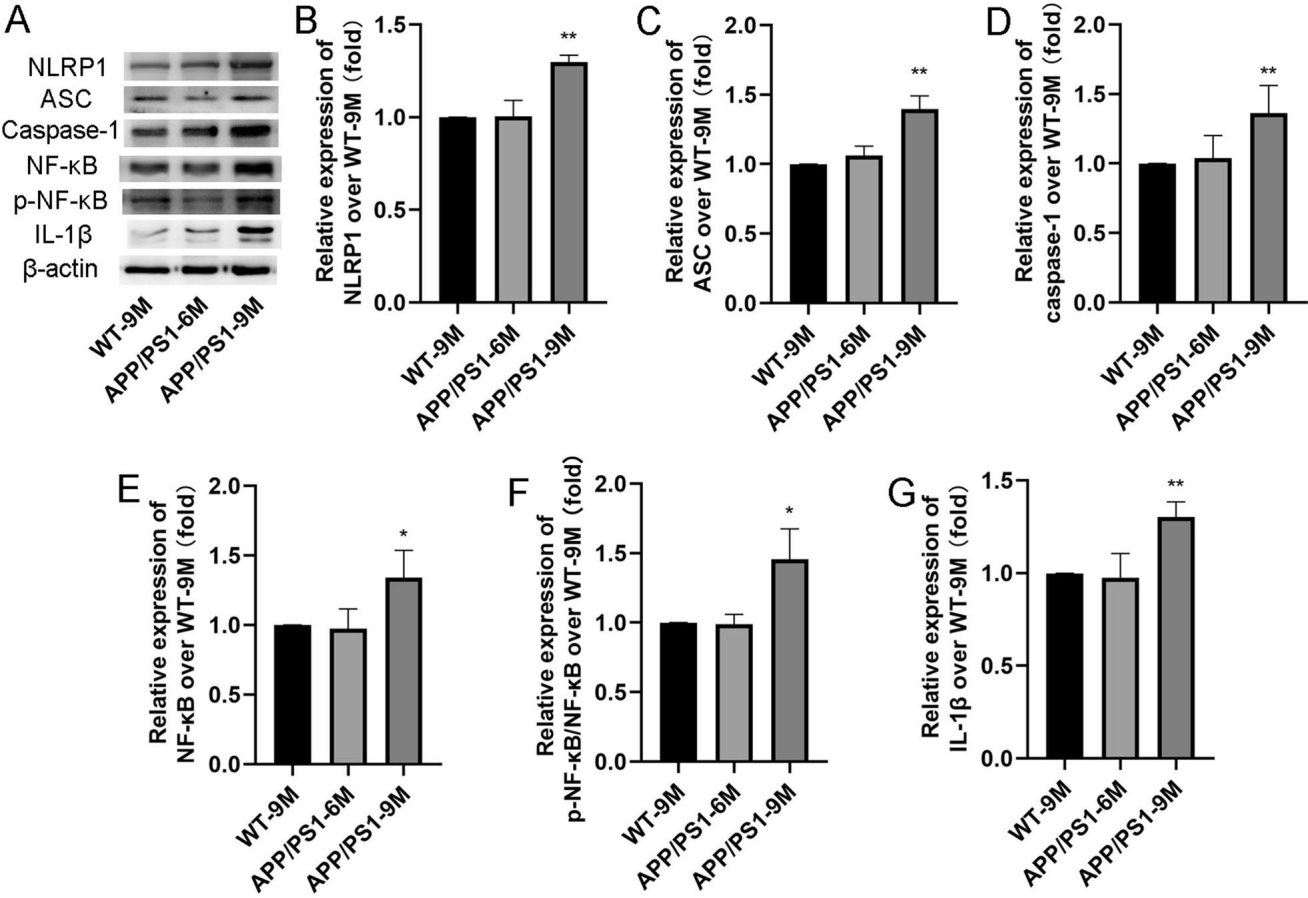
Effects of aging on NLRP1 inflammasome expression in APP/PS1 mice. **A** The bands of NLRP1, ASC, caspase-1, NF-κB, p-NF-κB, IL-1β and β-actin (Western blot). **B** The relative expression of NLRP1 over WT-9 M; **C** The relative expression of ASC over WT-9 M; **D** The relative expression of caspase-1 over WT-9 M; **E** The relative expression of NF-κB over WT-9 M; **F** The relative expression of p-NF-κB over WT-9 M; **G** The relative expression of IL-1β over WT-9 M. Results are expressed as mean ± SD. n = 4. *P < 0.05, **P < 0.01 vs WT-9 M group

Neuroinflammation is involved in the whole pathogenesis of AD, and studies have shown that NLRP1 is significantly increased in the brain of AD [13]. Therefore, we further detected the expression of NLRP1 inflammasome-related proteins of NLRP1, ASC, caspase-1, NF-κB, p-NF-κB and IL-1β. The results showed that, compared with the WT-9 M mice, there was no significant elevation in the levels of NLRP1, ASC, caspase-1, NF-κB, p-NF-κB and IL-1β in APP/PS1-6 M mice (Fig. 2A–G), while in APP/PS1-9 M mice, the levels of NLRP1, ASC, caspase-1, NF-κB, p-NF-κB/NF-κB and IL-1β were significantly increased (Fig. 2B: F(2,9) = 8.478; Fig. 2C: F(2,9) = 6.167; Fig. 2E: F(2,9) = 7.349; Fig. 2F: F(2,9) = 14.42; Fig. 2G: F(2,9) = 58.39; *P* < 0.05 or* P* < 0.01). These results suggested that NLRP1 inflammasome-mediated neuroinflammation is closely involved in Aβ generation and neuronal damage in the APP/PS1-9 M mice.

### Effects of aging on AMPK/mTOR pathway-mediated autophagy dysfunction in the APP/PS1 mice

**Fig. 3:**
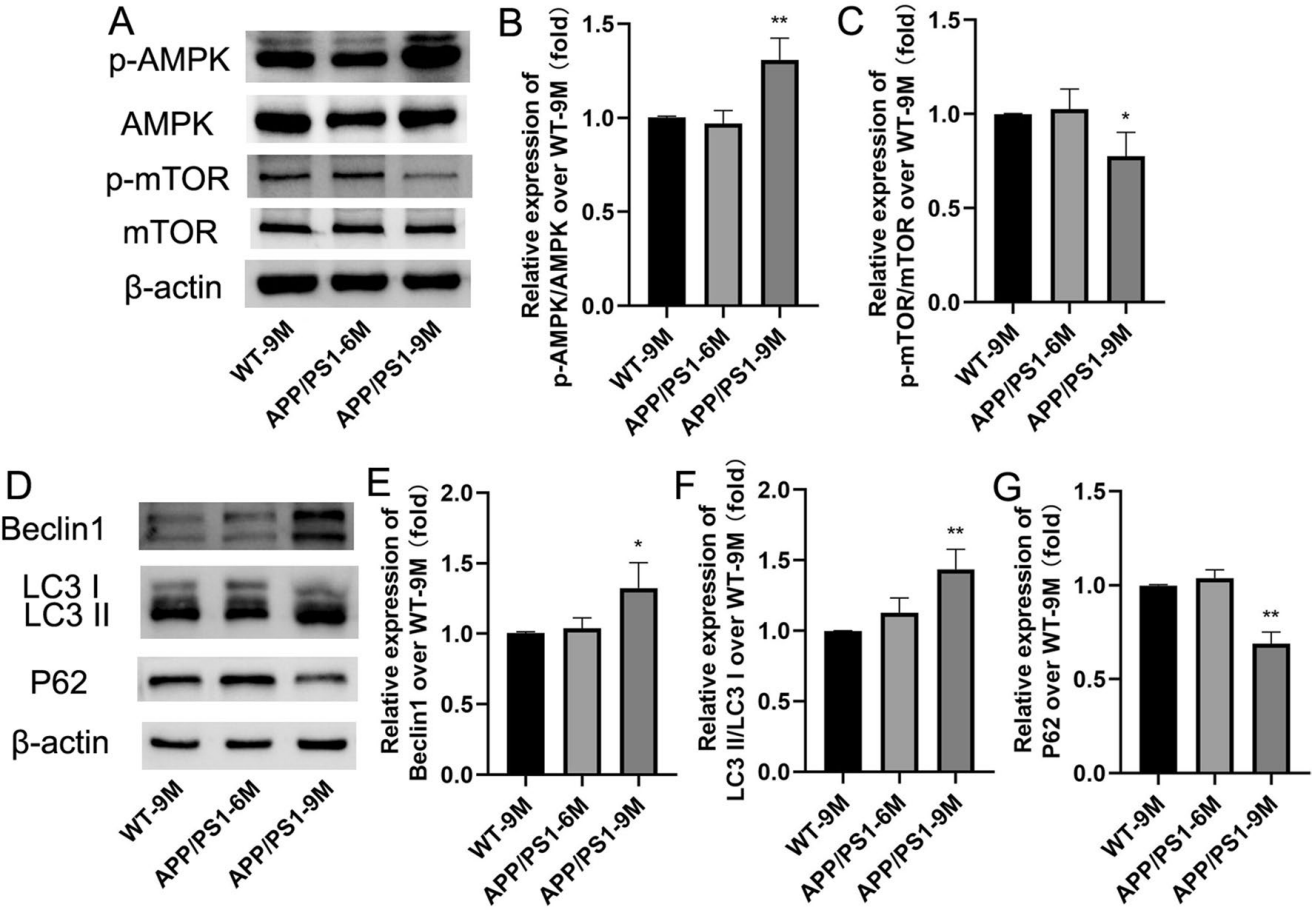
Effects of aging on AMPK/mTOR pathway-mediated autophagy in APP/PS1 mice. **A** The bands of AMPK, p-AMPK, mTOR, p-mTOR and β-actin (Western blot); **B** The relative expression of p-AMPK/AMPK over WT-9 M; **C** The relative expression of p-mTOR/mTOR over WT-9 M. **D** The bands of Beclin1, LC3, P62 and β-actin (Western blot); **E** The relative expression of Beclin1 over WT-9 M; **F** The relative expression of LC3II/LC3I over WT-9 M; **G** The relative expression of P62 over WT-9 M. Results are expressed as mean ± SD. n = 4, *P < 0.05, **P < 0.01 vs WT-9 M group

The downstream mechanisms of neuroinflammation are diverse, and autophagy has been considered as one of the most probable physiological regulatory pathways [33]. Increasing evidence has confirmed that the AMPK/mTOR pathway plays a vital role in regulating autophagy and neuronal damage [34]. Therefore, we further detected the changes of autophagy-related proteins of Beclin1, LC3, P62 and AMPK/mTOR pathway-related proteins of AMPK, p-AMPK, mTOR and p-mTOR. The results showed that, compared with the WT-9 M mice, there was no significant changes in the AMPK/mTOR pathway and autophagy in APP/PS1-6 M mice (Fig. 3A–G), while in APP/PS1-9 M, the levels of p-AMPK/AMPK, Beclin1 and LC3II/LC3I were significantly increased (Fig. 3B: F(2,9) = 17.64; Fig. 3E: F(2,9) = 7.349; Fig. 3F: F(2, 9) = 14.42; *P* < 0.05 or* P* < 0.01) and the levels of p-mTOR/mTOR and P62 were significantly decreased (Fig. 3C: F(2, 9) = 6.167; Fig. 3G: F(2, 9) = 58.39; *P* < 0.05 or* P* < 0.01). These results suggested that the AMPK/mTOR pathway mediated-autophagy function is significantly boosted in the brain of APP/PS1-9 M mice.

### NLRP1-siRNA treatment ameliorates abnormal behaviors in APP/PS1 mice

In the physiological and pathological changes of APP/PS1 mice, the NLRP1 inflammasome seems to act as an important leader to induce the autophagy dysfunction and Aβ deposition. Therefore, we transfected a LV-NLRP1-siRNA in 6 M APP/PS1 mouse for 12 weeks to knockdown the NLPR1 inflammasome. The results showed that treatment with lentivirus for 12 weeks showed obvious expression of GFP in hippocampus CA1 and cortex, indicating successful lentivirus infection in cortex and hippocampus in APP/PS1 mice (Additional file 3: Fig. S3).

**Fig. 4:**
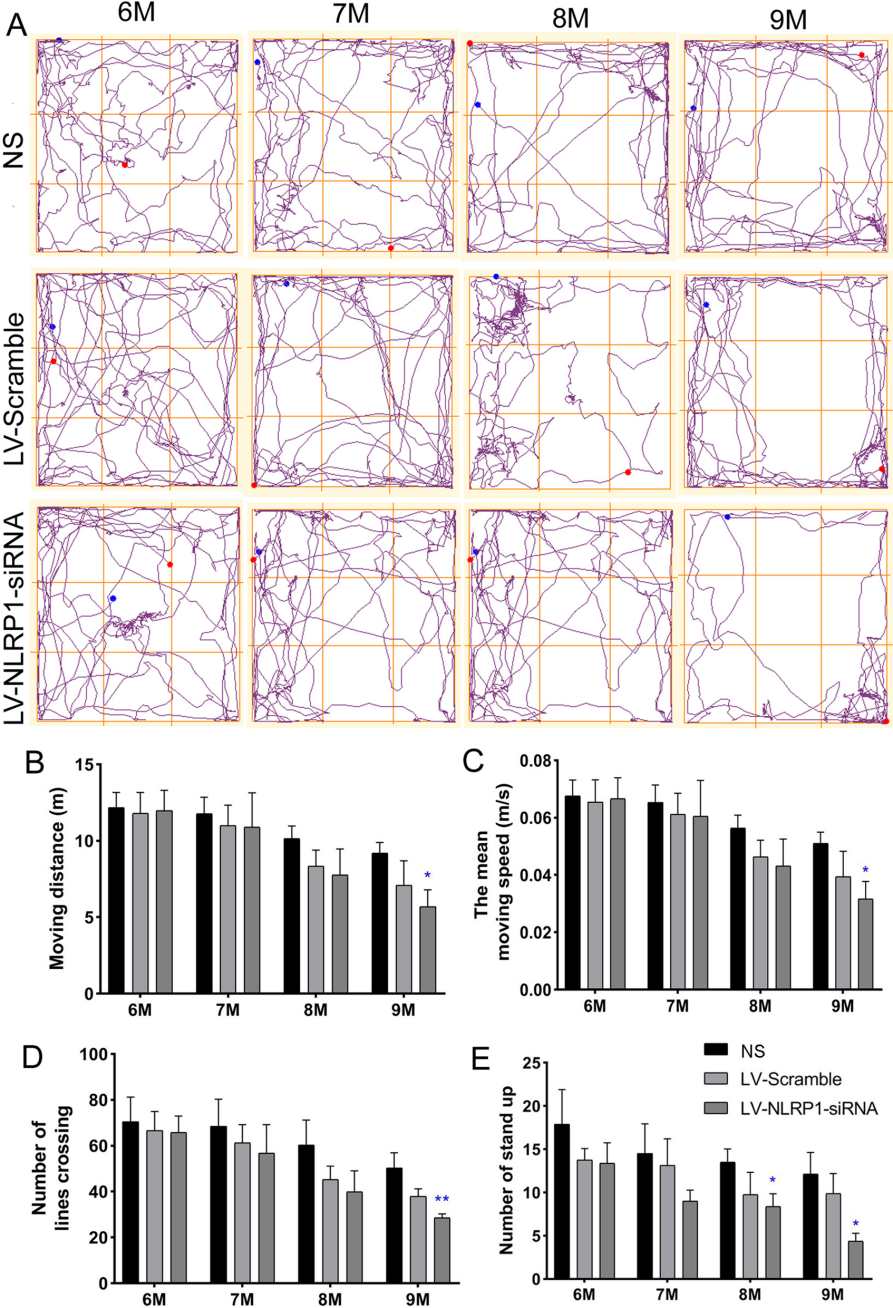
NLRP1-siRNA treatment improves abnormal motor behaviors in APP/PS1 mice (open field test, OFT). **A** The track of OFT from 6 to 9 M in APP/PS1 mice; **B** The moving distance (m); **C** The mean moving speed (m/s); **D** The number of lines crossing; **E** The number of stand up. Results are expressed as mean ± SD, n = 8. *P < 0.05, **P < 0.01 vs NS group

To confirm the effect of NLRP1 inflammasome activation on learning and memory impairments in APP/PS1 mice, we firstly examined the effect of NLRP1-siRNA treatment on motor behavior in APP/PS1 mice by using the OFT. The results showed that there were no significant differences in motor behavior among the NS, LV-scramble and LV-NLRP1-siRNA groups at 6 M before treatment. While compared with NS group, the moving distance (m) (Fig. 4B: F(3,21) = 4.324; P < 0.05), the mean moving speed (m/s) (Fig. 4C: F(3,21) = 4.329; P < 0.05)and the number of lines crossing (Fig. 4D: F(3,21) = 11.42; P < 0.01)were significantly decreased in the LV-NLRP1-siRNA group at 9 M, and the number of stand up (Fig. 4E: F(3,21) = 6.736; F(3,21) = 2.237; P < 0.05) were significantly decreased in the LV-NLRP1-siRNA group at 8 M and 9 M. The data suggested that NLRP1-siRNA treatment significantly decreases the motor behavior which has been reported to be significantly increased in APP/PS1 mice.

**Fig. 5:**
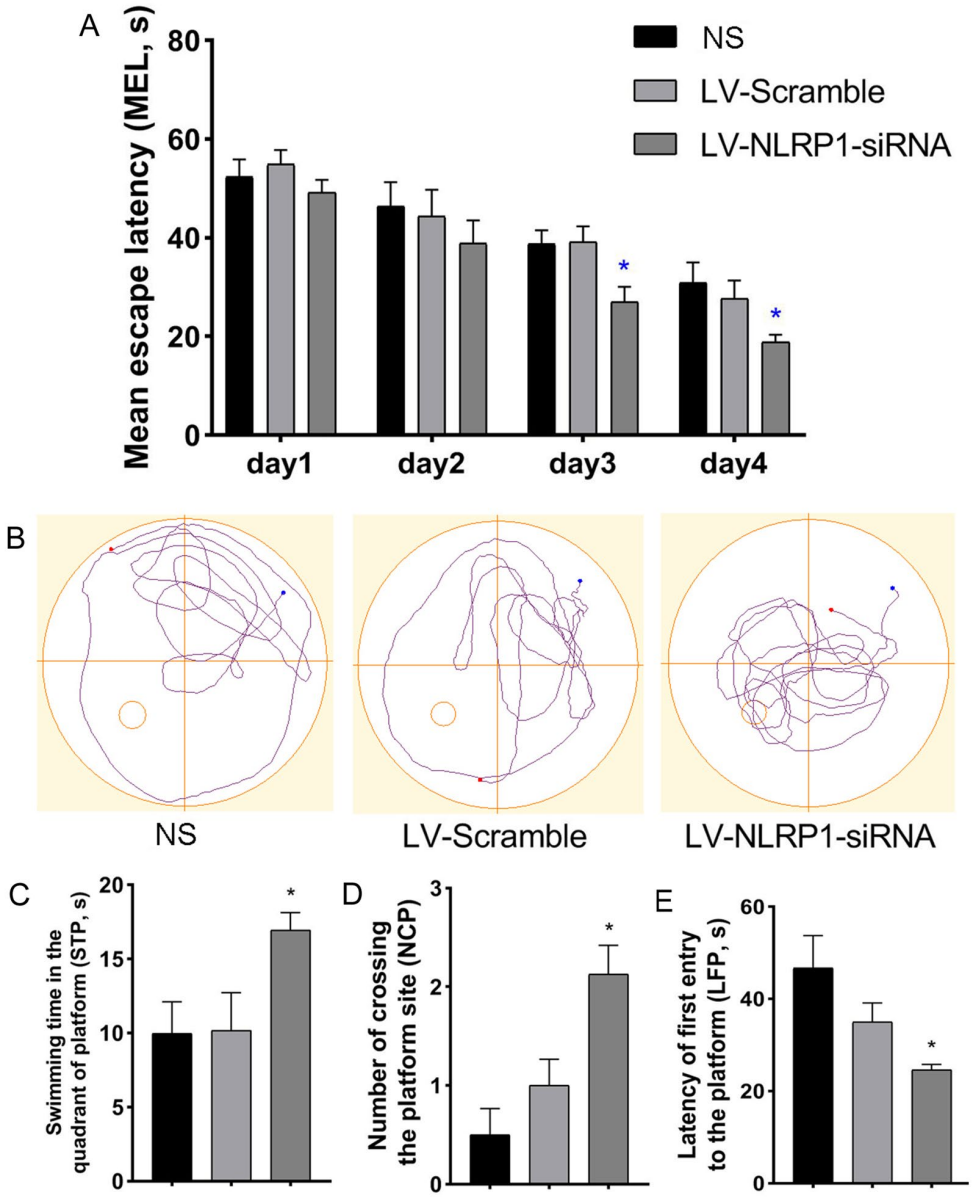
NLRP1-siRNA treatment ameliorates learning and memory impairments in APP/PS1 mice (Morris water maze, MWM). **A** The mean escape latency (MEL, s) in the orientation navigation test; **B** The track of MWM in APP/PS1 mice in the probe test; **C** The swimming time in the quadrant of platform (STP, s); **D** The number of crossing the platform (NCP); **E** The latency of first entry to the platform (LFP, s). Results are expressed as mean ± SD, n = 8. *P < 0.05 vs NS group

We also use the MWM test to detect the effect of NLRP1-siRNA treatment on learning and memory impairments in the APP/PS1 mice. The results of training trials showed that the escape latency had no significant difference in the first day (D1) and D2 among the NS, LV-scramble and LV-NLRP1-siRNA treatment groups. However, compared with the NS control group, the mean escape latency (MEL, s) was significantly reduced in the D3 and D4 in the NLRP1-siRNA treatment group (Fig. 5A: F(3,21) = 17.42; F(3,21) = 29.54;* P* < 0.05). On the fifth day of the probe test, the results showed that, compared with the NS control group, NLRP1-siRNA treatment could significantly increase the swimming time in quadrant of platform (STP, s) (Fig. 5D: F(2,21) = 3.790;* P* < 0.05) and the number of crossing platform (NCP) (Fig. 5D: F(2,21) = 9.039;* P* < 0.05) and decrease the latency of first entry to the platform (LFP, s) (Fig. 5D: F(2,21) = 5.349;* P* < 0.05) in APP/PS1 mice. The results indicated that NLRP1-siRNA treatment significantly alleviates the learning and memory impairments in APP/PS1 mice.

### NLRP1-siRNA treatment alleviates neuronal damage in APP/PS1 mice

**Fig. 6:**
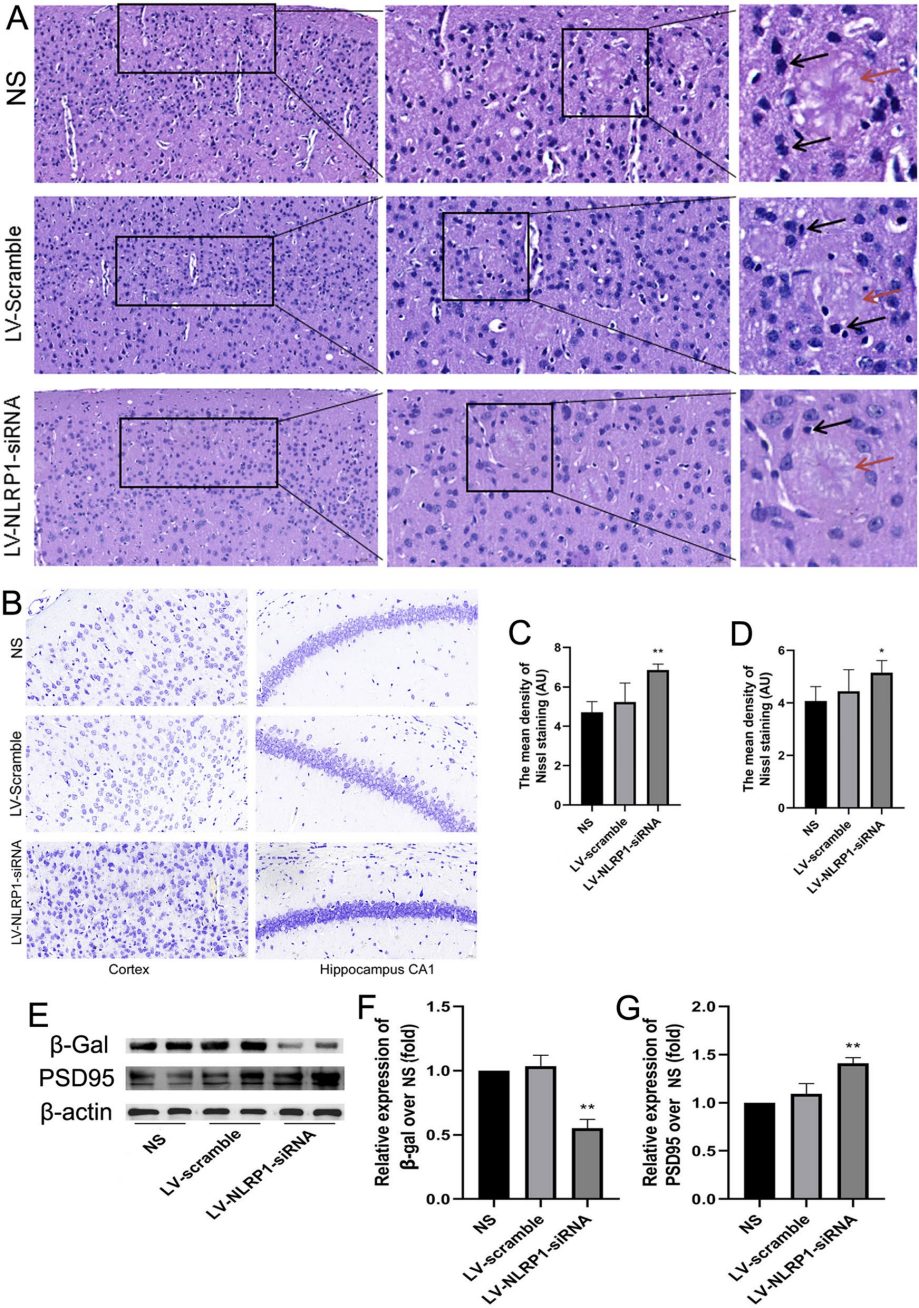
NLRP1-siRNA treatment alleviates neuronal damage in APP/PS1 mice. **A** The results of H&E staining (Cortex 200× , bar = 50 μm; 400× , bar = 20 μm). The black arrow indicates neuronal damage caused by Aβ deposition; The red arrow indicates Aβ plaque. **B** The results of Nissl staining in the cortex and hippocampus CA1 (400× , bar = 20 μm); (**C**–**D**) The mean density of Nissl bodies in the cortex and hippocampus CA1. **E** The bands of β-Gal, PSD95 and β-actin (Western blot); **F** The relative expression of β-Gal over NS; **G** The relative expression of PSD95 over NS. Results are expressed as mean ± SD, n = 4. ^*^P < 0.05, ^**^P < 0.01 vs NS group

We further used the H&E and Nissl staining to observe the effect of NLRP1-siRNA treatment on neuronal damage in APP/PS1 mice. The H&E results showed that there were significant neuronal damages and amyloid plaque in the cortex in NS and LV-scramble control group (Fig. 6A). Compared with the NS control group, NLRP1-siRNA treatment could significantly alleviate the neuronal damages, such as pyknosis and hyperchromatism (black arrows) and amyloid plaque (red arrows) in the cortex (Fig. 6A). The Nissl staining results also showed that NLRP1-siRNA treatment could significantly increase the Nissl bodies in the cortex and hippocampal CA1 regions compared with the NS control group (Fig. 6C: F(2,9) = 11.17; Fig. 6D: F(2,9) = 3.041; *P* < 0.05 or *P* < 0.01). To confirm the protective effect of NLRP1-siRNA treatment on neuronal damage in APP/PS1 mice, we further detected the expression levels of β-gal and PSD95 by immunoblot. The results showed that NLRP1-siRNA treatment could significantly decrease the expression of β-gal which was significantly increased in NS- and LV-scramble-treated APP/PS1 mice (Fig. 6C: F(2,9) = 72.87; *P* < 0.01). And NLRP1-siRNA treatment significantly increased the expression of PSD95 compared with NS or LV-scramble control group (Fig. 6G: F(2,9) = 37.08; *P* < 0.01). These results indicated that NLRP1 knockdown can alleviate neuronal damage and synaptic dysfunction in APP/PS1 mice.

### NLRP1-siRNA treatment reduces Aβ deposition in APP/PS1 mice

**Fig. 7:**
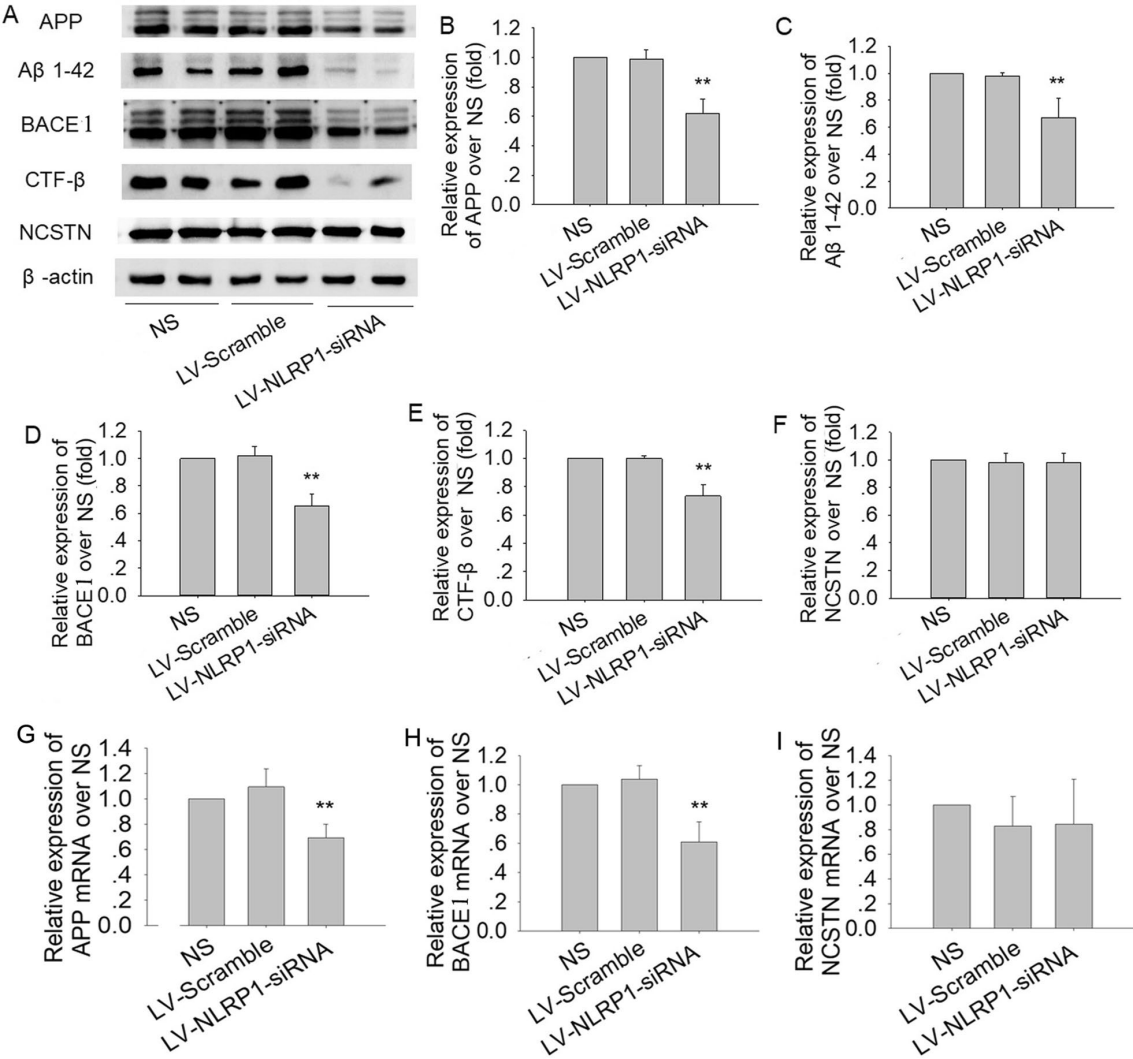
NLRP1-siRNA treatment reduces Aβ generation in APP/PS1 mice (Western blot). **A** The bands of APP, Aβ_1-42_, BACE1, CTF-β, NCSTN and β-actin; **B** The relative expression of APP over NS; **C** The relative expression of Aβ_1-42_ over NS; **D** The relative expression of BACE1 over NS; **E** The relative expression of CTF-β over NS; **F** The relative expression of NCSTN over NS. **G** The relative mRNA expression of APP (q-PCR); **H** The relative mRNA expression of BACE1 (q-PCR); **I** The relative mRNA expression of NCSTN (q-PCR). Results are expressed as mean ± SD, n = 4. ^*^P < 0.05, ^**^P < 0.01 vs NS

We further observed the effects of NLRP1 knockdown on Aβ generation and deposition in APP/PS1 mice. The immunoblot results showed that NLRP1-siRNA treatment could significantly decrease the expressions of APP, Aβ_1-42_, CTF-β and BACE1 (Fig. 7B: F(2,9) = 42.34; Fig. 7C: F(2,9) = 22.98; Fig. 7D: F(2,9) = 60.09;*P* < 0.01), and had no effect on NCSTN expression (Fig. 7A and F) compared with the NS group or LV-scramble group. The q-PCR results also showed that NLRP1-siRNA treatment significantly decreased the APP and BACE1 mRNAs (Fig. 7G: F(2,9) = 16.61; Fig. 7H: F(2,9) = 24.79;, *P* < 0.01), and did not affect NCSTN mRNA expression (Fg. 7I).

**Fig. 8:**
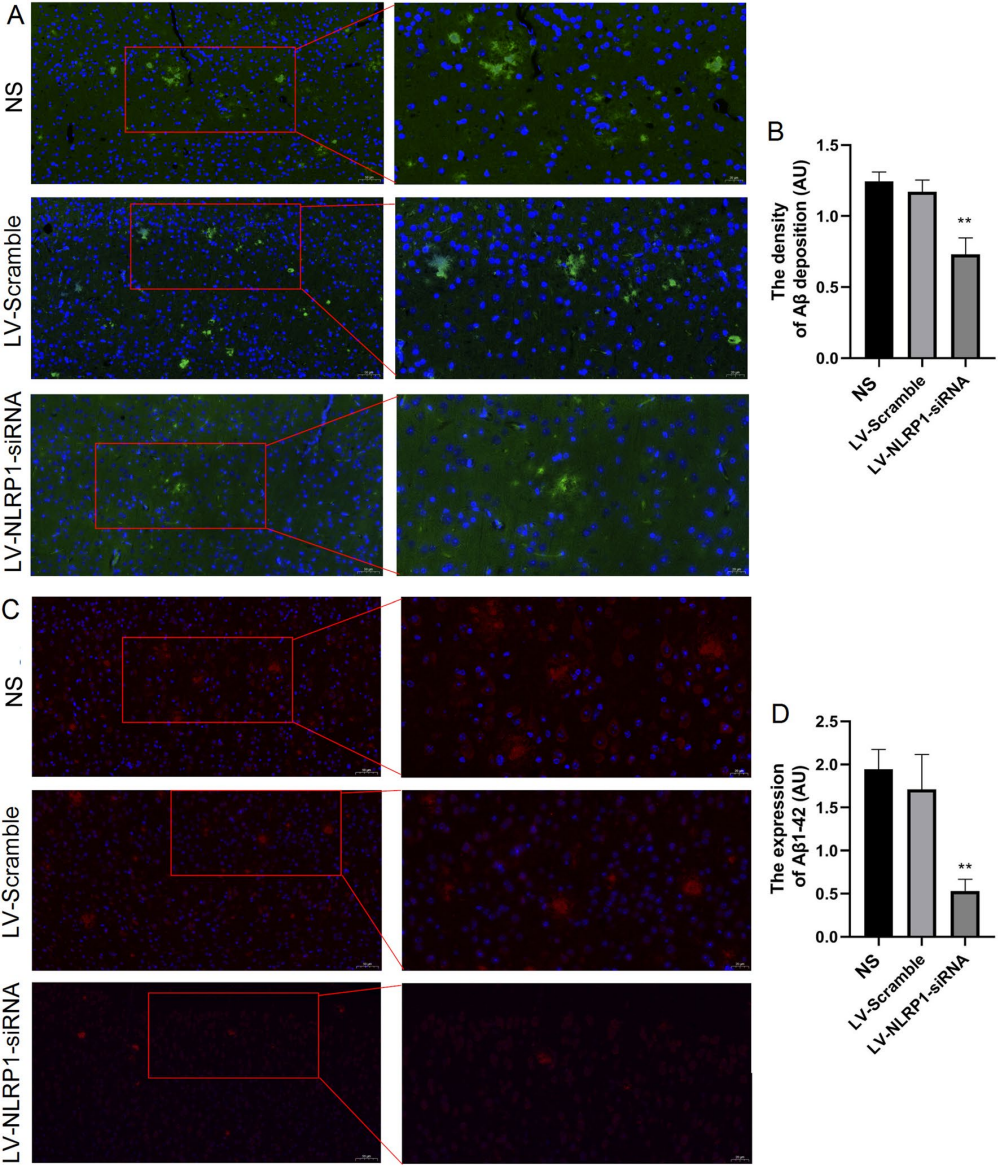
NLRP1-siRNA treatment reduces Aβ deposition in APP/PS1 mice. **A** The Aβ deposition in cortex (Thioflavin-S staining, 200× , bar = 50 μm; 400× , bar = 20 μm, exposure time: 200 ms); **B** The density of Aβ deposition. **C** The expression of Aβ_1-42_ in cortex (immunofluorescence 200× , bar = 50 μm; 400× , bar = 20 μm, exposure time:150 ms); **D** The expression of Aβ_1-42_. Results are expressed as mean ± SD, n = 4. ^**^P < 0.01 vs NS group

Additionally, we further detected the Aβ deposition by Thioflavin-S staining and the expression of Aβ_1-42_ by immunofluorescence. The results showed that, compared with the NS group, NLRP1-siRNA treatment significantly decreased the Aβ deposition in the cortex of APP/PS1 mice (Fig. 8A and B: F(2,9) = 9.396,* P* < 0.01). And similar to the Thioflavin-S staining, NLRP1-siRNA treatment significantly decreased the expression of Aβ_1-42_ (Fig. 8C and D: F(2,9) = 7.321;* P* < 0.01)_._ We can also observe similar results in lower magnification images of staining (Additional file 4: Fig. S4). These results suggested that NLRP1 knockdown can inhibit Aβ generation and deposition in APP/PS1 mice.

### NLRP1-siRNA treatment inhibits NLRP1 inflammasome activation in APP/PS1 mice

**Fig. 9:**
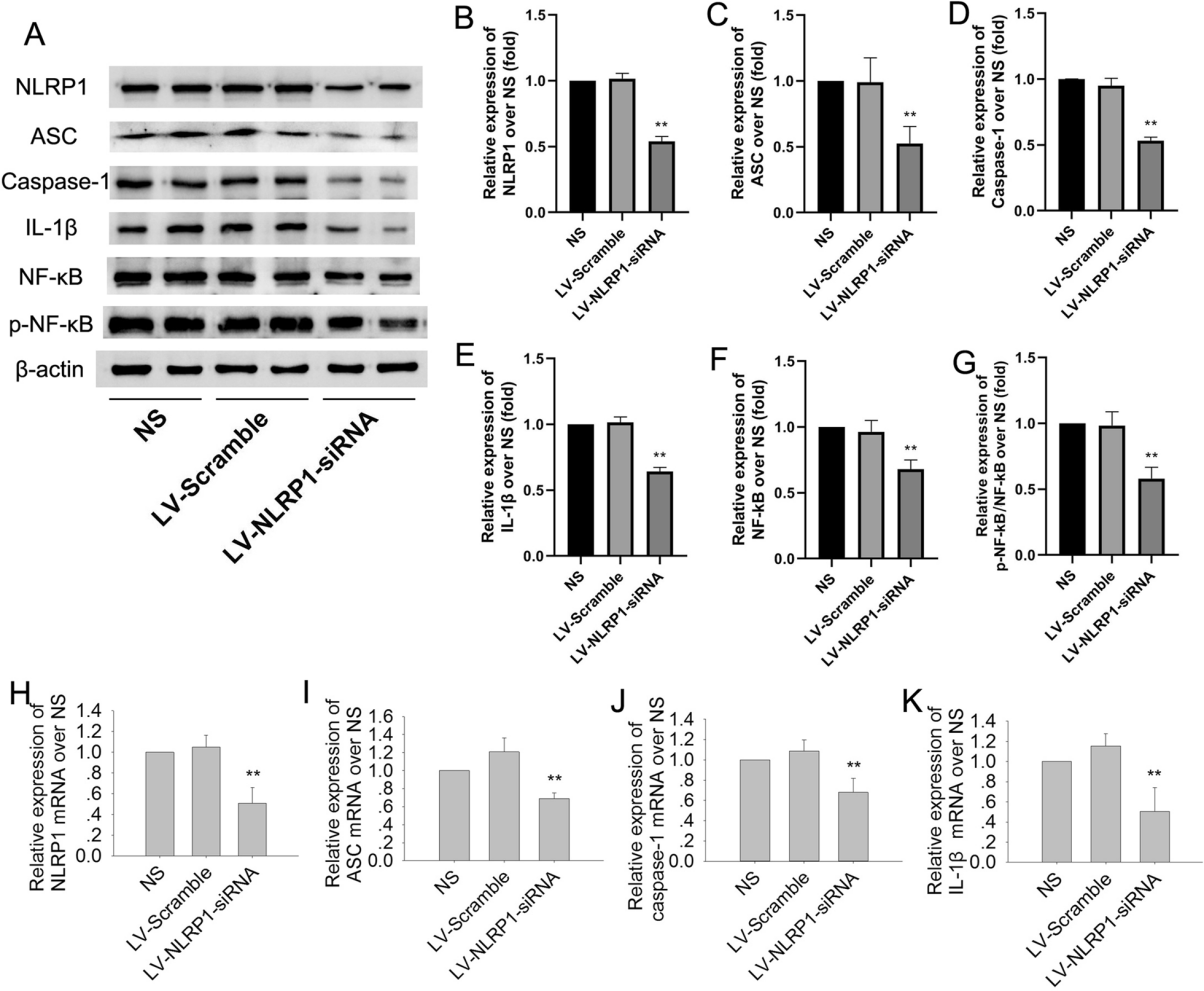
NLRP1-siRNA treatment inhibits NLRP1 inflammasome activation in APP/PS1 mice. **A** The bands of NLRP1, ASC, caspase-1, IL-1β, NF-κB, p-NF-κB and β-actin (Western blot); **B** The relative expression of NLRP1 over NS; **C** The relative expression of ASC over NS; **D** The relative expression of caspase-1 over NS; **E** The relative expression of IL-1β over NS. **F** The relative expression of NF-κB over NS; **G** The relative expression of p-NF-κB over NS. **H** The relative mRNA expression of NLRP1 (q-PCR); **I** The relative mRNA expression of ASC (q-PCR); **J** The relative mRNA expression of Caspase-1 (q-PCR); **K** The relative mRNA expression of IL-1β (q-PCR). Results are expressed as mean ± SD, n = 4. ^*^P < 0.05, ^**^P < 0.01 vs NS group

We further detected the effect of NLRP1-siRNA treatment on the expressions of NLRP1-related proteins and mRNAs. The results showed that the NLRP1-related proteins and mRNAs had no significant differences between the NS and LV-scramble groups. While compared with NS, NLRP1-siRNA treatment could significantly decrease the proteins and mRNAs of NLRP1, ASC, caspase-1 and IL-1β in APP/PS1 mice (Fig. 9B: F(2,9) = 299.2; Fig. 9C: F(2,9) = 16.99; Fig. 9D: F(2,9) = 199.8; Fig. 9E: F(2,9) = 207.0; Fig. 9H: F(2,9) = 29.95; Fig. 9I: F(2,9) = 28.75; Fig. 9J: F(2,9) = 17.65; Fig. 9K: F(2,9) = 19.53; *P* < 0.01). We also detected the levels of NF-κB and p-NF-κB. The results showed that, compared with NS or LV-scramble treatment group, NLRP1-siRNA treatment could significantly decrease the levels of NF-κB and p-NF-κB/NF-κB in APP/PS1 mice (Fig. 9F: F(2,9) = 30.01; Fig. 9G: F(2,9) = 37.10; *P* < 0.01). These results suggested that NLRP1 knockdown can significantly ameliorate the neuroinflammation in APP/PS1 mice.

### NLRP1-siRNA treatment reverses AMPK/mTOR pathway-mediated autophagy dysfunction in APP/PS1 mice

**Fig. 10:**
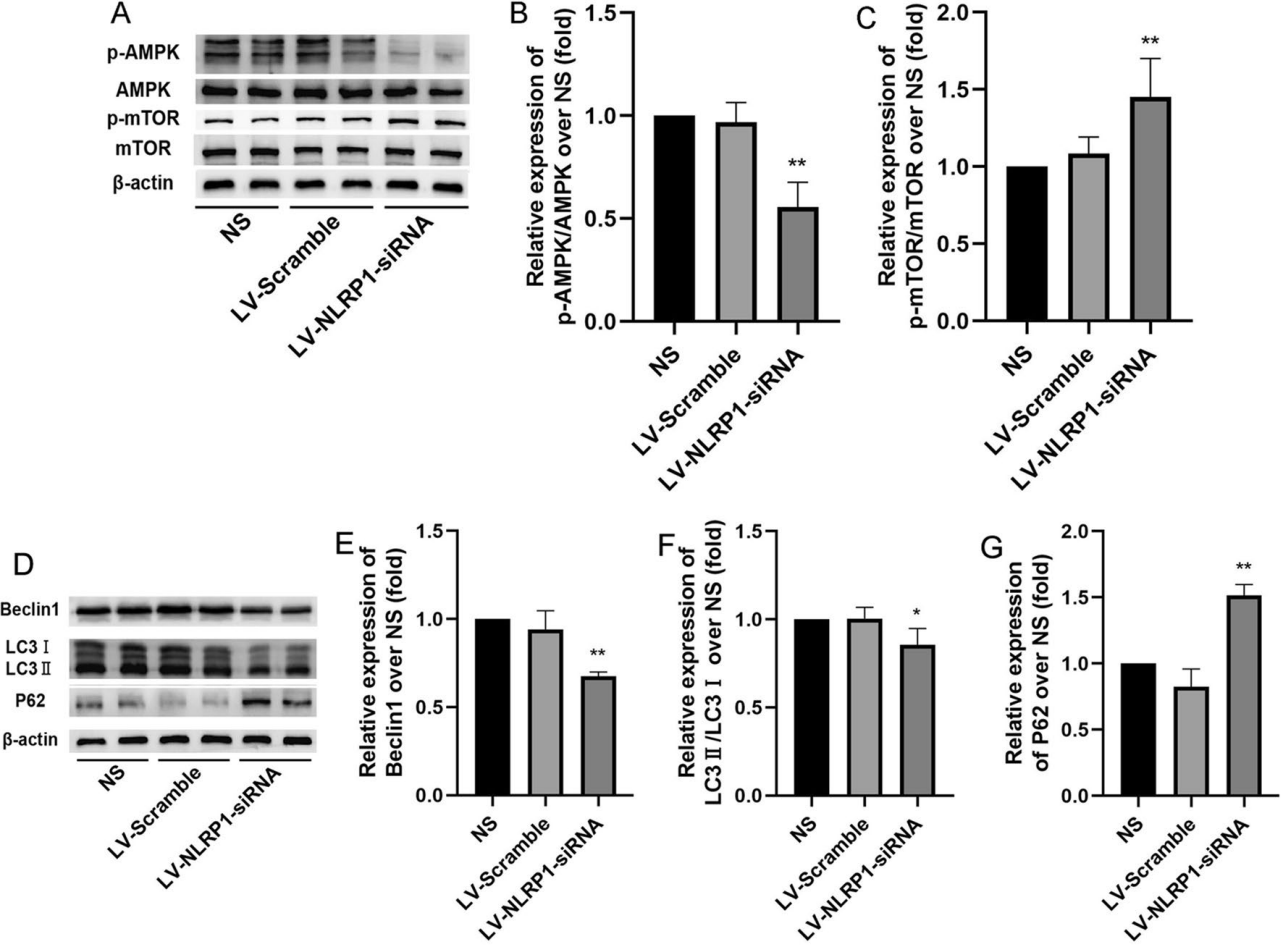
NLRP1-siRNA treatment reverse AMPK/mTOR pathway mediated autophagy dysfunction in APP/PS1 mice (Western blot). **A** The bands of AMPK, p-AMPK, mTOR, p-mTOR and β-actin; **B** The relative expression of p-AMPK/AMPK over NS; **C** The relative expression of p-mTOR/mTOR over NS. **D** The bands of Beclin1, LC3, P62 and β-actin; **E** The relative expression of Beclin1 over NS; **F** The relative expression of LC3II/LC3I over NS; **G** The relative expression of P62 over NS. Results are expressed as mean ± SD, n = 4. ^*^P < 0.05, ^**^P < 0.01 vs NS group

We further investigated the effect of NLRP1-siRNA treatment on the autophagy and AMPK/mTOR pathway in APP/PS1 mice. The results showed that there were no significant differences in the autophagy and AMPK/mTOR pathway between the NS and LV-scramble groups. While compared with NS, NLRP1-siRNA treatment significantly decreased the levels of p-AMPK/AMPK, Beclin1 and LC3II/LC3I(Fig. 10b: F(2,9) = 31.09; Fig. 10E: F(2,9) = 31.35; Fig. 10F: F(2,9) = 6.774; *P* < 0.05 or* P* < 0.01), and increased the levels of p-mTOR/mTOR and P62 in APP/PS1 mice (Fig. 10C: F(2,9) = 9.537; Fig. 10G: F(2,9) = 63.35; *P* < 0.01). These results suggested that NLRP1 knockdown can reverse AMPK/mTOR pathway mediated-autophagy dysfunction in APP/PS1 mice.

## Discussion

Growing studies have demonstrated a connection between NLRP1 inflammasome and progression of AD, although the mechanism has not been fully elucidated [12, 35]. The present study indicated that NLRP1 inflammasome activation and autophagy dysfunction are closely implicated in Aβ generation and deposition during AD progression. Meanwhile, we found that knockdown of NLRP1 could significantly improve learning and memory impairments, alleviate Aβ generation and deposition and inhibit NLRP1 inflammasome activation in 9 M APP/PS1 mice. The results also demonstrated that knockdown of NLRP1 significantly reversed the AMPK/mTOR pathway and inhibited the autophagy function in APP/PS1 mice. Our results suggested that inhibition of NLRP1 inflammasome activation might reverse the AMPK/mTOR pathway-mediated autophagy dysfunction, resulting in reduction of Aβ generation and deposition and ameliorating the pathological process of AD.

In recent years, neuroinflammation has been recognized as a potential driver of neurodegenerative diseases, such as AD and Parkinson’s disease (PD) [36, 37]. Although inflammation is a defensive response in nature, chronic inflammation can induce tissue damage and even death. In AD, neuroinflammation is considered to be reaction and contributor to neuronal damage and also plays an important role in the pathogenesis of AD [38]. It has been reported that increased IL-1β in AD brains contributes to AD pathology by increasing APP gene expression and Tau hyperphosphorylation [39]. The inflammasomes, multiprotein complexes in cytoplasm, play important roles for the recruitment and activation of caspase-1, which promotes the maturation of pro-inflammatory cytokines, such as IL-1β and IL-18 [40]. Inflammasomes play a critical role in regulating the inflammatory response and contribute to the pathogenesis of various neurologic diseases [41]. High-level NOD-like receptor protein 1 (NLRP1) inflammasome was found to be expressed in the central nervous system, especially in neurons [42]. The NLRP1 inflammasome was reported to be activated in AD patients [35]. Inhibition of NLRP1 expression could ameliorate age-related cognitive deficits [14]. Moreover, the inflammasome adaptor protein of ASC (apoptosis-associated speck-like protein containing a caspase recruitment domain) contributes to the spread of inflammation and forms a complex with Aβ to promote inflammation [43]. Therefore, we firstly confirmed the relationship between NLRP1 inflammasome activation and Aβ generation during the progress of AD. The present study indicated that, compared with WT control group, the expressions of both Aβ generation-related proteins, such as APP, BACE1 and Aβ_1-42_, and NLRP1 inflammasome-related proteins, such as NLRP1, ASC, caspase-1 p-NF-κB and IL-1β, were significantly increased in 9 M APP/PS1 mice, but had no significant changes in 6 M APP/PS1 mice. We further knockdown the expression of NLRP1 through LV-NLRP1-siRNA to confirm the effect of NLRP1 on regulation of Aβ generation. We found that NLRP1-siRNA treatment not only significantly decreased the expression of NLRP1, but also significantly reduced ASC, caspase-1, p-NF-κB and IL-1β expression. We speculated that knockdown of NLRP1 may be attenuate neuronal inflammation and damage in the brain of APP/PS1 mice, thus resulting in downregulation of the NLRP1 inflammasome-related proteins. The specific mechanism may need further study. Meanwhile, knockdown of NLRP1 also significantly reduced the expressions of APP, CTF-β, BACE1 and Aβ_1-42_ proteins and mRNAs. Furthermore, NLRP1-siRNA treatment significantly decreased the Aβ deposition and Aβ1-42 expression in cortex of 9 M APP/PS1 mice. The results suggested that knockdown of NLRP1 can decrease Aβ generation. Meanwhile, we found that inhibition of NLRP1 significantly improved learning and memory impairments, and alleviated neuronal damage and Aβ deposition in cortex of APP/PS1 mice. Our data suggested that NLRP1 inflammasome closely involves in Aβ generation and deposition in the progression of AD. However, the mechanism of NLRP1 inflammasome in regulating Aβ generation remains incompletely understood.

Autophagy is the major self-degradative process in the cell, through which the autophagosomes transport cytoplasmic materials, such as misfolded proteins and damaged organelles, to the lysosomes for cellular renovation and homeostasis [44]. In healthy mammalian cells, there is a low basal level of autophagy to dilute noxious components [45]. Many stresses such as nutrient starvation and inflammation can enhance autophagic activity, which closely involves cell death, tumor suppression and removal of microorganisms [46]. It has been reported that autophagy plays a key role in regulating Aβ generation and clearance [20]. On the one hand, Aβ peptides can generate during autophagic turnover of APP-riched organelles in the autophagosomes [20, 21, 47]. On the other hand, the maturation of autophagolysosomes and their regressive passage from dystrophic and degenerating neurites toward the neuronal body are blocked in AD [47]. This leads to a massive accretion of autophagic vacuoles in neurons and is correlated to dysfunction of autophagy in AD. However, autophagy may be a double-edged sword in progression of AD and has controversial functions [48, 49]. It has been reported that lysosome is an important organelle for Aβ generation in autophagic vacuoles after autophagy activation [22]. And the immature autophagic vacuoles were significantly accumulated in AD brains and APP/PS1 mice, which maybe an important source of Aβ production [50]. However, more studies reported that inhibition of autophagy could decrease Aβ clearance and induce intracellular aggregation of Aβ, which contributed to neurodegeneration in AD [48]. In the present study, we found that the formation of Aβ was significantly increased along with the disorder of autophagy in 9 M APP/PS1 mice, which was manifested by increased expressions of Beclin1 and LC3-II/LC3-I, reduced expression of p62 and activation of its upstream pathway in 9 M APP/PS1 mice. Our study also showed that knockdown NLRP1 significantly decreased Aβ generation and deposition along with a significant decrease of autophagy in 9 M APP/PS1 mice. These data suggested that inhibition of NLRP1 inflammasome can decrease autophagy function, which may also contribute to decreasing of Aβ production, and resulting in improvement of AD.

The AMP-activated protein kinase (AMPK) and mammalian target of rapamycin (mTOR) are two main nutrient-sensing pathways in response to stress. The AMPK/mTOR pathway plays important roles in modulation of autophagy [51]. The AMPK is an energy sensor, which can promote autophagy to regulate cellular metabolism and homeostasis. The mTOR is a serine/threonine protein kinase, which can inhibit autophagy to modulate cell growth, proliferation and survival [52]. AMPK can activate autophagy through decreasing the activity of mTOR. In this study, we found that the expression of p-AMPK was significantly upregulated and the p-mTOR was significantly downregulated in brain tissues of 9 M APP/PS1 mice, suggesting that the AMPK/mTOR pathway-mediated autophagy is closely associated with the progression of AD. The Beclin 1, an autophagy-related protein, regulates the formation of autophagosomes. The LC3-I can combine with the phosphatidylethanolamine (PE) to further generate LC3-II. The autophagosomal membranes can recruit LC3-II after fusion of autophagosomes to lysosomes to play degradation [53]. P62 is one of selective cargo receptors, which interact with LC3-II in recognition and selective autophagy to degenerate misfolded proteins [54]. Our results also indicated that the expressions of Beclin 1 and LC3-II were significantly increased and P62 was significantly decreased in the brain tissues of 9 M APP/PS1 mice. Meanwhile, we found that knockdown of NLRP1 significantly decreased the level of p-AMPK, Beclin 1 and LC3 II, and increased the level of p-mTOR and P62 in the brain tissues of 9 M APP/PS1 mice. These data suggested that neuroinflammation and autophagy might interact each other to promote progression of AD.

The relationship between neuroinflammation and autophagy has been previously reported [55, 56]. However, whether NLRP1 inflammasome participates in regulation of autophagy during progression of AD is still unclear. It has been reported that the APP/PS1 mice showed higher levels of IL-1β along with increased autophagic vesicles in dystrophic neurons. And the levels of inflammatory mediators closely related to expression of mTOR and Beclin-1 [57]. The mTOR signaling was significantly inhibited and the level of Beclin-1 was positively correlated with the levels of IL-1β and TNF-α in hippocampus and cortex in APP/PS1 mice, suggesting that neuroinflammation could induce autophagy in AD [57]. This is consistent with our results, in which the expression of IL-1β decreased by NLRP1-siRNA treatment, and the expression of Beclin-1 also decreased. A recent study reported that, in H_2_O_2_-induced trophoblast cells, the NLRP1 inflammasome was significantly activated along with an excessive autophagy, while the NLRP1 activator inhibited autophagy and NLRP1-siRNA increased autophagy [58]. In another study, in primary cultured rat cortex neurons, oxygen–glucose deprivation and re-oxygenation (OGD/R) caused excessive autophagy, the ratio of LC3-II/LC3-I and the expression of Beclin 1 were significantly increased, and the p62 expression was significantly decreased. And the propofol treatment significantly inhibited autophagy and improved OGD/R-induced neuronal damage [59]. In the present study, we found that, compared with WT 9 M mice, the NLRP1 inflammasome activation and the autophagy level were not significantly increased and along with lower levels of Aβ production and APP in APP/PS1 6 M mice. While in APP/PS1 9 M mice, we found a significant increase of NLRP1 inflammasome and autophagy levels along with higher levels of Aβ production and APP. Meanwhile, we found that knockdown of NLRP1 significantly decreased the activation of NLRP1 inflammasome and autophagy in brain tissues along with significant decrease of Aβ production and APP, and significantly improved the learning and memory impairments in APP/PS1 9 M mice. Our data suggested that NLRP1 inflammasome and autophagy dysfunction play important roles in regulation of Aβ production. Therefore, we speculated that NLRP1 inflammasome activation may enhance the autophagy function, which can engulf the excessive APP and increase Aβ generation in AD progression. And inhibition of NLRP1 inflammasome may decrease the autophagy function, thereby reducing Aβ generation and deposition in 9 M APP/PS1 mice. However, it is still not completely clear how NLRP1 inflammasome and autophagy interact each other, and further researches are still needed to elucidate the mechanism.

All in all, our results indicated that NLRP1 inflammasome activation and AMPK/mTOR mediated-autophagy dysfunction are closely implicated in Aβ generation and deposition in APP/PS1 9 M mice. Meanwhile, we found that knockdown of NLRP1 significantly improved learning and memory impairments, Aβ generation and autophagy dysfunction in APP/PS1 9 M mice. Our study suggested that NLRP1 and autophagy might be important targets to delay the progression of AD. However, the NLRP1 inflammasome and autophagy are complex and involve a series of signaling pathways [60]. And the mechanism of NLRP1 inflammasome and autophagy in progression of AD remains unknown and needs further elucidation in vitro and in vivo.

## Supplementary Information

**Additional file 1: Fig. S1.** Experimental flow chart.

**Additional file 2: Fig. S2**. Effects of aging on neuronal damage and Aβ deposition in APP/PS1 mice. (A) The changes of pathomorphology in cortex of WT-9 M, APP/PS1-6 M and -9 M mice (H&E staining, 400 × , bar = 50 μm, n = 4); (B) The changes of Aβ deposition in cortex of WT-9 M, APP/PS1-6 M and -9 M mice (Thioflavin-S staining, 400 × , bar = 20 μm, n = 4). The black arrows indicate pyknotic cells around Aβ plaques and the red arrows indicate Aβ deposition in cortex.

**Additional file 3: Fig. S3**. Effect of lentivirus treatment for 12 weeks on GFP expression in the hippocampus CA1 and cortex in APP/PS1 mice (400 × , n = 4, bar = 50 μm).

**Additional file 4: Fig. S4**. NLRP1-siRNA treatment reduces Aβ deposition in cortex of APP/PS1 mice (Thioflavin-S staining and Aβ_1-42_ by immunofluorescence, 100 × , bar = 100 μm).

**Additional file 5: Table S1.** Primary antibodies used in Immunoblot analysis studies.

**Additional file 6: Table S2**. Primers used in qPCR studies.

## Data Availability

All data generated or analysed during this study are included in this published article.
