# Supplementary figures and images for: Inhibition of NLRP1 inflammasome improves autophagy dysfunction and Aβ disposition in APP/PS1 mice
Source: Behav Brain Funct. 2023 Apr 13;19:7. doi: 10.1186/s12993-023-00209-8 (PMC10100229; doi:10.1186/s12993-023-00209-8)

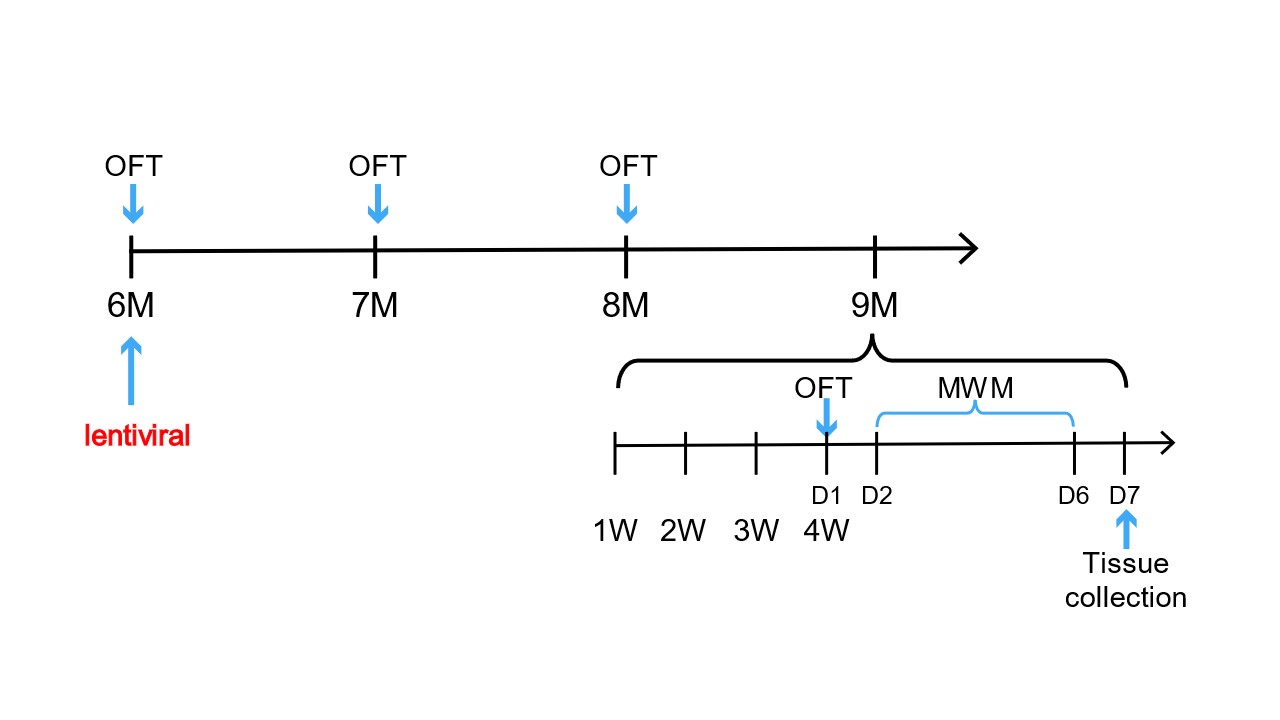

Supplement: Supplementary file 1 — Additional file 1: Fig. S1. Experimental flow chart. [file 12993_2023_209_MOESM1_ESM.jpg]

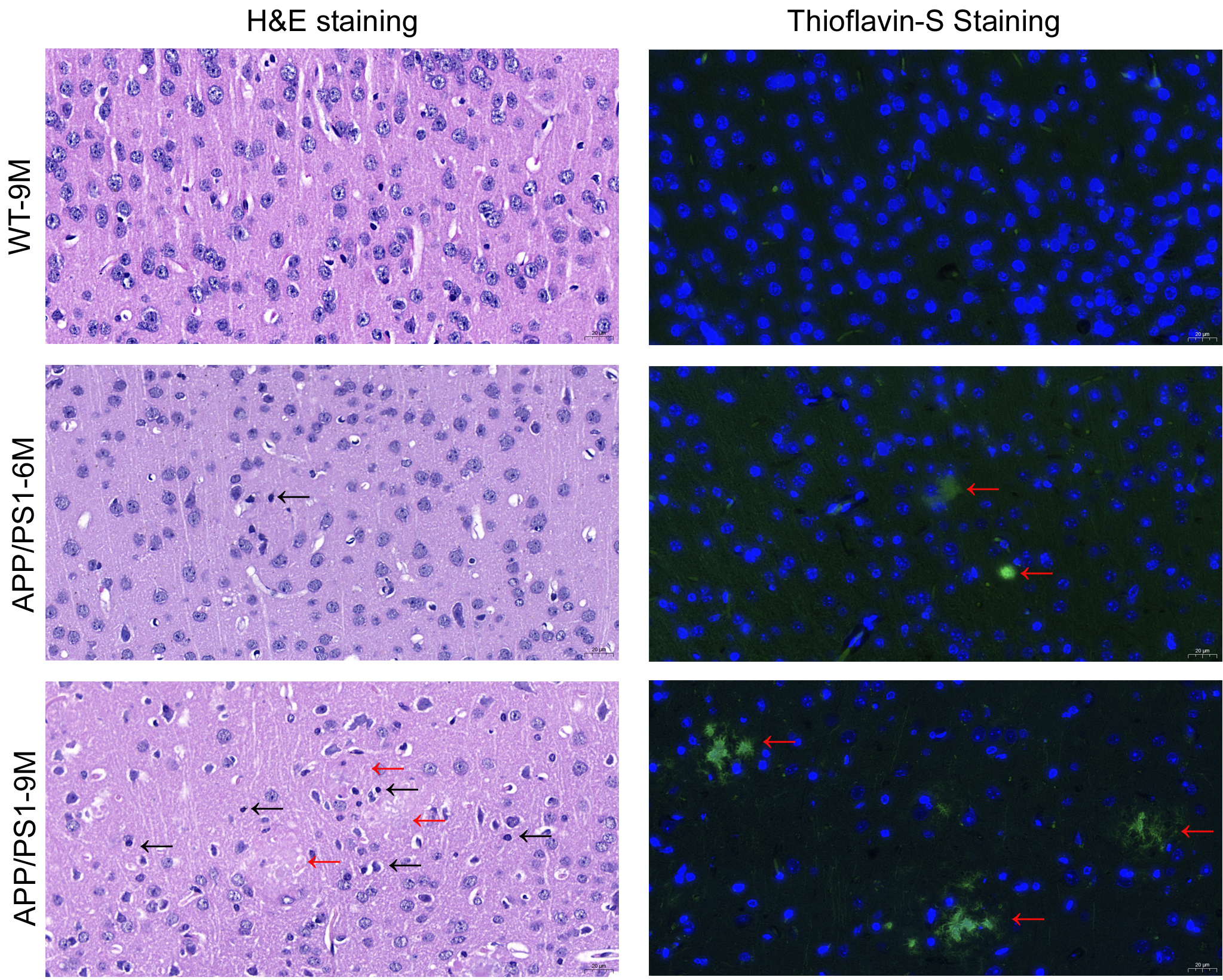

Supplement: Supplementary file 2 — Additional file 2: Fig. S2. Effects of aging on neuronal damage and Aβ deposition in APP/PS1 mice. (A) The changes of pathomorphology in cortex of WT-9 M, APP/PS1-6 M and -9 M mice (H&E staining, 400 × , bar = 50 μm, n = 4); (B) The changes of Aβ deposition in cortex of WT-9 M, APP/PS1-6 M and -9 M mice (Thioflavin-S staining, 400 × , bar = 20 μm, n = 4). The black arrows indicate pyknotic cells around Aβ plaques and the red arrows indicate Aβ deposition in cortex. [file 12993_2023_209_MOESM2_ESM.jpg]

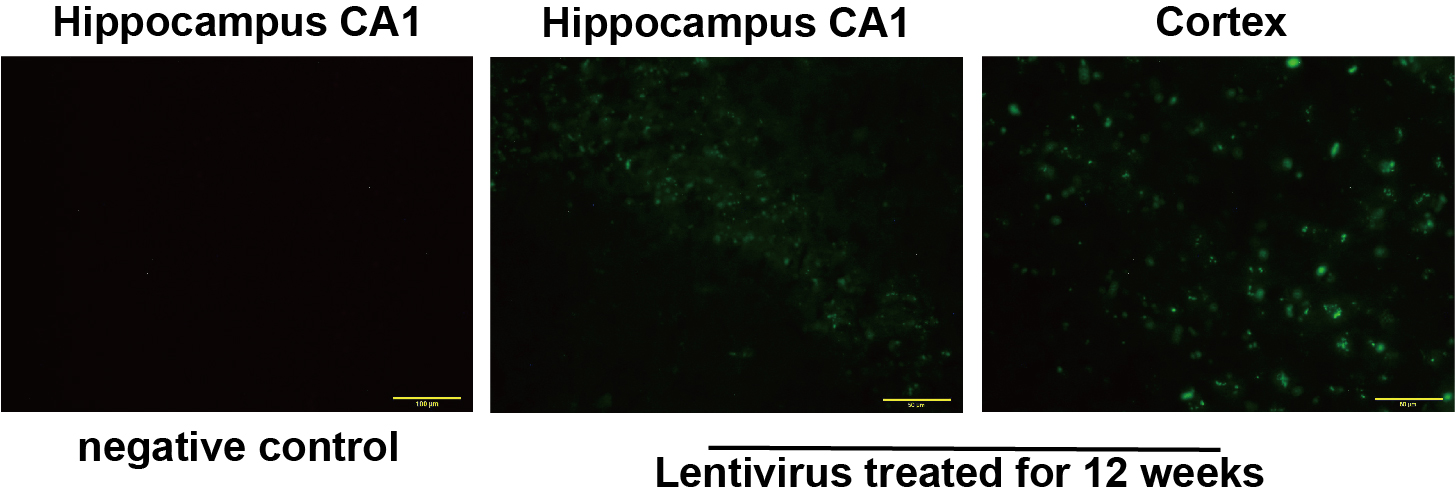

Supplement: Supplementary file 3 — Additional file 3: Fig. S3. Effect of lentivirus treatment for 12 weeks on GFP expression in the hippocampus CA1 and cortex in APP/PS1 mice (400 × , n = 4, bar = 50 μm). [file 12993_2023_209_MOESM3_ESM.jpg]
