# Supplementary material for: Inhibition of NLRP1 inflammasome improves autophagy dysfunction and Aβ disposition in APP/PS1 mice
Source: Behav Brain Funct. 2023 Apr 13;19:7. doi: 10.1186/s12993-023-00209-8 (PMC10100229; doi:10.1186/s12993-023-00209-8)
Supplement: Supplementary file 5 — Additional file 5: Table S1. Primary antibodies used in Immunoblot analysis studies. [file 12993_2023_209_MOESM5_ESM.docx]

**Table S1.** Primary antibodies used in Immunoblot analysis.

| **Target** | **Company and Item** | **Dilution ratio** |
| --- | --- | --- |
| **NLRP1** | Abcam, ab3683 | 1:2000 |
| **ASC** | Bioss Technology, bs-6741R | 1:500 |
| **Caspase-1** | Abcam, ab1872 | 1:1000 |
| **IL-1β** | Abcam, ab9722 | 1:1000 |
| **NF-κB** | Servicebio Technology, GB11142 | 1:1000 |
| **p-NF-κB** | Servicebio Technology,GB11142-1 | 1:1000 |
| **APP** | Affinity biosience, DF6012 | 1:2000 |
| **BACE1** | Affinity biosience, DF7594 | 1:1500 |
| **NCSTN** | Affinity biosience, DF6242 | 1:1000 |
| **Aβ1-42** | Abcam, ab201060 | 1:2000 |
| **PSD95** | Servicebio Technology, GB11277 | 1:1000 |
| **β-gal** | Wanlei-bio, WL03124 | 1:500 |
| **AMPK** | Affinity, AF6423 | 1:1000 |
| **p-AMPK** | Bioworld Technology, bs-4010 | 1:1000 |
| **mTOR** | Proteintech Group, 66888-1-lg | 1:5000 |
| **p- mTOR** | Bioworld Technology, bs-4706 | 1:1000 |
| **Beclin1** | Wanleibio, WL02508 | 1:1000 |
| **P62** | Wanleibio, WL02385 | 1:500 |
| **LC3** | Proteintech Group, 14600-1-AP | 1:1000 |
| **β-Actin** | Servicebio Technology, GB12001 | 1:2000 |
