# Supplementary material for: Inhibition of NLRP1 inflammasome improves autophagy dysfunction and Aβ disposition in APP/PS1 mice
Source: Behav Brain Funct. 2023 Apr 13;19:7. doi: 10.1186/s12993-023-00209-8 (PMC10100229; doi:10.1186/s12993-023-00209-8)
Supplement: Supplementary file 6 — Additional file 6: Table S2. Primers used in qPCR studies. [file 12993_2023_209_MOESM6_ESM.docx]

**Table S2.** Primers used in q-PCR studies.

| Target | Forward primer (5’-3’) | Reverse primer (5’-3’) |
| --- | --- | --- |
| NLRP1 | TGGCACATCCTAGGGAAATC | TCCTCACGTGACAGCAGAAC |
| ASC | GTCACAGAAGTGGACGGAGTG | CTCATCTTGTCTTGGCTGGTG |
| Caspase-1 | CGTGGAGAGAAACAAGGAGTG | AATGAAAAGTGAGCCCCTGAC |
| IL-1β | CTGCTTCCAAACCTTTGACC | AGCTTCTCCACAGCCACAAT |
| APP | GGCCCTCGAGAATTACATCA | GTTCATGCGCTCGTAGATCA |
| BACE1 | TTTGTGGAGATGGTGGACAA | TACACACCCTTTCGGAGGTC |
| NCSTN | CTGACCACTCTGGCTCCTTC | GCTGCTGAAGTTGGTTCCTC |
| β-Actin | GATTACTGCTCTGGCTCCTAGC | GACTCATCGTACTCCTGCTTGC |
